# Detecting stigmatizing language with large language models: mind the settings

**DOI:** 10.1093/jamiaopen/ooag037

**Published:** 2026-04-02

**Authors:** Teenu Xavier, Jane M Carrington, Joshua Lambert W

**Affiliations:** School of Nursing, George Mason University, Fairfax, VA 22030, United States; College of Nursing, University of Florida, FL 32603, United States; College of Nursing, University of Cincinnati, Cincinnati, OH 45221, United States

**Keywords:** large language models, stigmatizing language, clinical notes, natural language processing, clinical documentation

## Abstract

**Background:**

Stigmatizing language in clinical documentation can contribute to healthcare disparities and affect patient–provider relationships. Given their strong capacity for contextual language understanding, large language models (LLMs) offer potential for detecting and reducing such language. This study evaluates the accuracy of LLMs in detecting stigmatizing language, focusing on model size, temperature settings, and the inclusion of examples.

**Methods:**

We evaluated multiple configurations of 2 local Llama-based large language models, Llama 3.2 (3B) and Llama 3.1 (8B) with varying temperature (0.25, 0.5, 0.75) and the inclusion of exemple prompts. The models were evaluated on 3643 de-identified clinical notes obtained from a tertiary care teaching hospital. Performance was assessed using accuracy, True Positive Rate (TPR), and True Negative Rate (TNR), with human annotator performance used as a benchmark.

**Results:**

The 8B model with a temperature of 0.25 and examples achieved the highest overall accuracy (70.2%), with the best TPR (94.1%), but the lowest TNR (47.4%). The 3B model without examples achieved the highest TNR (99.7%) but a very low TPR (2%). The inclusion of examples improved model accuracy across all configurations, while temperature settings had a variable impact, with smaller models benefiting from higher temperatures and larger models performing better at lower temperatures. ED provider notes showed higher accuracy (69.4%) and the plan of care was the lowest (55.8%).

**Conclusion:**

Model size, temperature, and the inclusion of examples play a critical role in optimizing open-source LLM performance. Tailoring these parameters to note types enhances effectiveness. Further research should refine these models for broader clinical application and assess their potential to reduce bias in healthcare documentation.

## Introduction

Stigmatizing language in electronic health records (EHRs) is a significant concern as it can influence healthcare providers’ perceptions and treatment of patients, potentially contributing to bias in clinical decision-making.^1^ Such language whether it reflects disapproval of patient behaviors, doubts about the credibility of patient-reported symptoms, or broad, stereotype-driven characterizations with terms like “drug addict,” “non-compliant,” or “poor historian,” fostering higher stigma and negative attitudes. Over time, these patterns can perpetuate biases and influence care decisions often unconsciously, resulting in unequal treatment, misdiagnosis, and undertreatment in ways that ultimately compromise patient well-being.^2–9^ Stereotypes and assumptions (eg, about pain tolerance, compliance, or criminality) lead to disparities in pain management, cancer care, and preventive services.^7^^,^^10–12^ This risk is heightened by the growing reliance on EHRs as authoritative, objective sources of patient information. EHR documentation is shared, persistent, and often reused across clinical encounters; stigmatizing terms, once entered, can be copied forward and viewed by multiple providers, reinforcing implicit bias over time and across care teams.^1^ As a result, implicit biases embedded in clinical documentation may lead to unequal treatment practices, diminished patient trust,^13^ and ultimately compromised health outcomes for marginalized groups.^14^ Patients who sense judgment or doubt from their providers are less likely to adhere to treatment plans or to share sensitive health information, ultimately worsening health outcomes and patient satisfaction.^14^

Beyond individual patient–provider interactions, the implications extend to the use of advanced artificial intelligence (AI) tools applied to EHR data. As clinical text increasingly informs predictive models, treatment recommendations, and clinical triaging systems, uncorrected stigmatizing language may inadvertently encode and amplify biases in these computational frameworks.^15^ For instance, language that conveys moral judgment or suspicion may disproportionately affect Black patients, who already face systemic barriers to equitable care.^16^^,^^17^ Over time, these biases once encoded into the logic of AI tools can exacerbate health disparities, making it more challenging to break entrenched cycles of inequitable healthcare. By reducing biased language in clinical documentation, the healthcare community can create a more equitable environment, ensuring that patients receive compassionate, unbiased, and patient-centered care.

The rapid evolution of AI and machine learning has introduced powerful analytic tools such as large language models (LLMs) capable of processing and interpreting vast quantities of text.^18^^,^^19^ LLMs have demonstrated remarkable utility in a wide range of healthcare applications, including educational content generation,^20^^,^^21^ research support,^22^ EHR analysis,^23^ and specialized fields like psychiatry,^20^ oncology^24–26^ and cardiology.^27^^,^^28^ They also assist with tasks like drafting discharge summaries^29–31^ and medical journalism.^32^ With the ability to process language in context, LLMs can be trained to recognize stigmatizing terms and phrases and detect bias patterns in patient records. By analyzing both language content and tone, LLMs could offer critical insights for healthcare providers, helping to minimize unintended biases in documentation and promoting more equitable care.

Prior work has examined stigmatizing language in clinical documentation using lexicon-based methods,^33^ manual annotation,^12^ and traditional machine learning approaches,^34^ demonstrating that judgmental or biased language is prevalent and associated with disparities in care. However, these studies have largely focused on identifying or quantifying stigma rather than examining how emerging large language models behave under different analytic conditions. As LLMs are increasingly used for clinical text analysis, it is critical to understand sensitivity of LLMs to model configuration choices and variations in prompts that can lead to inconsistent outputs.^35^ This sensitivity is influenced by the specific wording, structure, and format of prompts,^35^^,^^36^ the type of model used,^37^ the type of data used, and the specific tasks they are applied to.^38^ Such sensitivities underscore the importance of carefully crafting prompts, establishing standardized coding frameworks, and ensuring methodological rigor when using LLMs to detect stigmatizing language in clinical texts. The present study addresses this gap by systematically evaluating LLM performance across model sizes, temperature settings, prompting strategies, and clinical note types, rather than proposing a new stigmatization detection model.

The primary goal was to evaluate the inherent capabilities of local, open source, non-fine-tuned LLMs in classifying stigmatizing language within clinical notes across various parameter choices. A unique consideration in this work is the clinical note type, which varies in purpose, audience, and linguistic conventions. For example, progress notes often contain daily impressions and informal shorthand, while discharge summaries may follow more structured formats and reflect longer-term clinical perspectives. These differences can affect both the presence and expression of stigmatizing language, as well as how well models detect it. As such, examining model performance across note types is essential for understanding LLM utility in real-world documentation settings. The objectives of the present study were to: (1) evaluate the accuracy of large language models (LLMs) in classifying stigmatizing versus non-stigmatizing language in clinical notes, (2) assess the impact of model size, temperature settings, and the inclusion of examples on the performance of LLMs in identifying stigmatizing language, (3) analyze the variation in model performance across different types of clinical notes, and (4) compare the performance of LLMs against manual validation providing a comprehensive assessment of LLM performance within this context.

## Methods

The data for this analysis were obtained from a large academic medical center. Electronic health record (EHR) data were extracted for adult patients with Hospital-Acquired Conditions (HACs), identified using ICD-10 codes for complications such as retained foreign objects after surgery, embolism, blood incompatibility, pressure ulcers, falls and trauma, catheter-associated urinary tract infections, vascular catheter-associated infections, poor glycemic control, surgical site infections, deep vein thrombosis, and iatrogenic pneumothorax. Inclusion criteria were patients aged 21 years or older with at least one documented HAC and a hospital length of stay of 3 or more days. These data were reused from a previously published study, and the research team granted permission to use them for this analysis.^38^

A total of 3643 deidentified clinical notes were assessed, with 1783 identified as containing stigmatizing language as identified by a clinical expert and 1860 classified as non-stigmatizing. The annotation process was carried out independently by 2 members of the research team to ensure reliability and rigor. Any disagreements in classification were reviewed and discussed, and when consensus could not be reached, a third team member adjudicated the final decision. The details of the process is highlighted in the study by Xavier et al.^34^ In this study, stigmatizing language is defined as language that unintentionally conveys negative meanings or reinforces social hierarchies and power imbalances, thereby perpetuating bias or discrimination toward certain individuals or groups.^1^ It often manifests subtly through the use of quotation marks to express disbelief, phrasing that questions credibility, or judgmental terms that imply blame or moral failure or stereotyping or labeling patients.^2^ This structured annotation process, designed to minimize bias and enhance consistency, was developed and applied as part of a previous study.^34^ The following paraphrased examples of stigmatized language illustrate the annotation approach used in this study:

*Example of stigmatizing language (paraphrased): “The patient refused to cooperate again despite multiple reminders.”*

*Example of non-stigmatizing language (paraphrased): “The patient declined the procedure after risks and benefits were discussed.”*

### Ethics approval and consent to participate

The study was exempt from review by the Institutional Review Board (IRB) and waived the need for consent to participate. All procedures followed the ethical principles outlined in the Declaration of Helsinki.

We selected open-source, locally deployable LLMs and commonly used parameter settings that reflect real-world experimentation by researchers and health systems. The clinical notes were analyzed to assess stigmatizing language using 2 different Llama local large language models (LLMs): a 3B-sized model (hugging-quants/Llama-3.2-3B-Instruct-Q8_0-GGUF) and an 8B-sized model (lmstudio-community/Meta-Llama-3.1-8B-Instruct-GGUF). The 3B and 8B denote the number of parameters, with the 3B model being smaller and generally faster but potentially less accurate than the larger 8B model. These models were used without any fine-tuning on clinical data, which is a common approach when leveraging pre-trained models for specific tasks.

The models were evaluated at different temperature levels (0.25, 0.5, and 0.75) to assess the impact of the randomness of the predictions by the model on classification accuracy. These settings were selected to span a practical range of generation variability and allowed us to examine how randomness influences classification consistency in a sensitive clinical task. Temperature is a hyperparameter that controls the randomness of the model’s predictions.^39^ Lower temperature typically makes the models more predictable and repetitive, while higher temperature typically makes the model more random and creative.^40^^,^^41^ Models were asked to return “N” for clinical text identified as not having stigmatizing language and “S” for clinical text deemed to have stigmatizing language. Given that the returned response by the model was supposed to be one character, the model’s response length was limited to a single token.

Models were either asked to classify with or without the 3 most previous examples of true stigmatizing language and 3 true examples of non-stigmatizing language within the prompt (6 total). The model without examples was selected to explore whether it could generalize the task effectively without explicit training data.

In summary, our primary analysis would classify the 3643 clinical notes using: 2 model sizes (3B, 8B), 3 temperature settings (0.25, 0.5, and 0.75), and with or without examples. Thus, resulting in 12 different combinations of model size, temperature, and examples. We also considered note type as an important source of variation in our analysis. Different types of clinical notes such as progress notes, discharge summaries, and admission notes can differ significantly in their structure, tone, and linguistic patterns. To account for this, we evaluated whether both the model’s classification performance and the presence of stigmatizing language varied by note type. This allowed us to understand better how the model performs within the diverse documentation landscape of clinical care. For configurations without examples, the prompt used was: “Please label the following sentence as ‘S’ if it contains stigmatizing language, and ‘N’ if it does not contain stigmatizing language.” For the example-based condition, the prompt construction included the same instruction followed by previous examples from the dataset. More specifically, the model received the previous 3 stigmatizing examples and 3 previous non-stigmatizing examples to guide classification. The dataset’s order via seeded randomness to ensure the rolling-window examples were independent. Our stateless rolling-window few-shot prompting strategy and seeded randomness was chosen to ensure reproducibility, as well determine robustness of the zero versus few-shot strategy across perturbed prompts. Additionally, in late January 2025, the new 8B Deepseek R1 reasoning model (lm studio-community/DeepSeek-R1-Distill-Llama-8B-GGUF) was employed over the same set of varying parameters to determine if a “chain-of-thought”/“reasoning” model could improve the overall performance. Deepseek model employ a reasoning step before outputting their answers. We applied the reasoning step implicitly by running DeepSeek-R1 with its default settings as the customization feature was not available when we did the analysis. We also used the same prompts as for the Llama 3.2 (3B) and Llama 3.1 (8B) models, in both the example-based and no-example conditions. This reasoning step takes more computation time and tokens, so therefore, fewer clinical notes were attempted (*n* = 300) due to time, and an unlimited number of tokens were allowed in the settings.

LM Studio 0.3.8 was used locally to download and run the models mentioned above via LM Studios server functionality locally (offline). A Windows PC with an i9-13900KF processor, featuring 24 cores operating at a base clock speed of 3.00 GHz, 64 GB of installed RAM, and an NVIDIA RTX 4090 GPU with 24 GB dedicated GPU memory.

## Results

The pre-trained models demonstrated varying levels of accuracy depending on the model size, temperature setting, and with and without examples (Table 1). The best performance in terms of overall accuracy was the 8B (largest) model with a temperature of 0.25 (smallest) with examples, which achieved an overall accuracy of 70.2%. This model had the best overall True Positive Rate (TPR; Predicting “Stigmatizing” as “Stigmatizing”) at 94.1% although it performed the worst overall True Negative Rate (TNR; Predicting “Not Stigmatizing” as “Not Stigmatizing”) at 47.4%. The worst performance in terms of overall accuracy was the 3B (smallest) model with a temperature of 0.25 (smallest) without examples, which achieved an overall accuracy of 51.9%. This model had the best overall True Negative Rate (TNR; Predicting “Not Stigmatizing” as “Not Stigmatizing”) at 99.7% although it performed the worst overall True Positive Rate (TPR; Predicting “Stigmatizing” as “Stigmatizing”) at 2%.

**Table 1.** Performance models in classifying stigmatizing vs non-stigmatizing language by model size, temperature, and example usage.

			Not stigmatizing *N* = 1860				Stigmatizing *N* = 1783				
Llama model	Temperature	Example used	Predicted correct		Predicted incorrect		Predicted correct		Predicted incorrect		Overall accuracy
			*N*	%	*N*	%	*N*	%	*N*	%	%
8B	0.25	True	881	47.4	979	52.6	1677	94.1	106	5.9	70.2
8B	0.5	True	890	47.8	970	52.2	1667	93.5	116	6.5	70.2
8B	0.75	True	890	47.8	970	52.2	1619	90.8	164	9.2	68.9
3B	0.75	True	1692	91.0	168	9.0	681	38.2	1102	61.8	65.1
3B	0.5	True	1718	92.4	142	7.6	620	34.8	1163	65.2	64.2
3B	0.25	True	1752	94.2	108	5.8	516	28.9	1267	71.1	62.3
8B	0.75	False	1812	97.4	48	2.6	325	18.2	1458	81.8	58.7
8B	0.5	False	1820	97.8	40	2.2	315	17.7	1468	82.3	58.6
8B	0.25	False	1829	98.3	31	1.7	271	15.2	1512	84.8	57.6
3B	0.75	False	1841	99.0	19	1.0	72	4.0	1711	96.0	52.5
3B	0.5	False	1847	99.3	13	0.7	55	3.1	1728	96.9	52.2
3B	0.25	False	1854	99.7	6	0.3	35	2.0	1748	98.0	51.9

Of the 12 overall variations, the 6 models without examples made up the bottom 6, the worst performers in terms of overall accuracy, although they were the top 6 performers in terms of TNR. The 6 models with examples had the highest 6 overall accuracies although the lowest 6 TNR. In terms of temperature, the 3B (smaller) model performed better with higher temperature (0.75) while the 8B (larger) model’s preference differed based on with or without examples.

Despite taking longer to run and having a supposed superior “reasoning” approach the Deepseek R1 8B model showed middling performance to the Meta Llama models, achieving approximately 60% accuracy in its best scenario (temperature = 0.25, with examples) on a subset of 300 notes.

**Figure 1.**
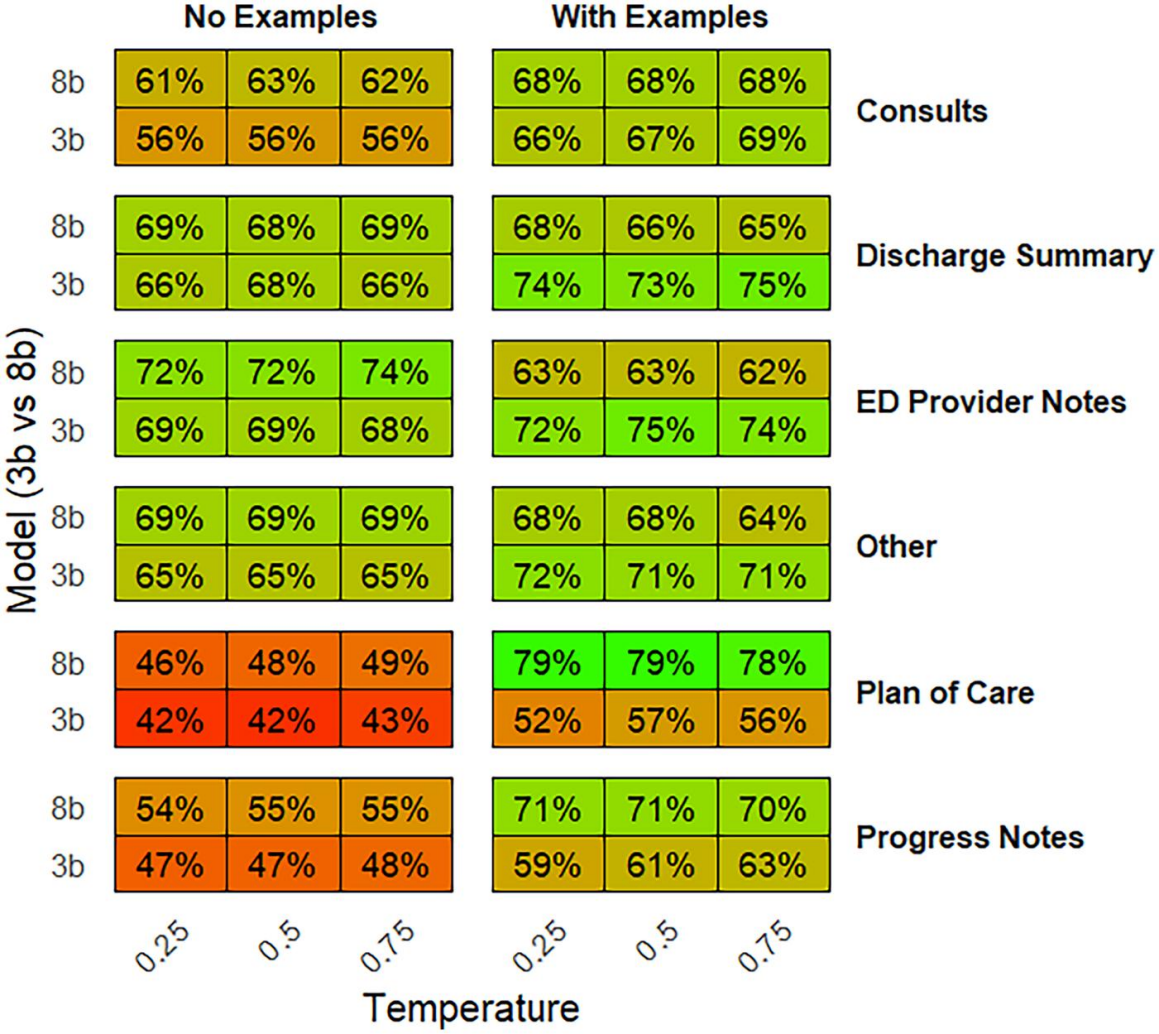
Performance by model, temperature, example use, and note type.

In this study, we also examined the accuracy of pre-trained models in predicting stigmatizing versus non-stigmatizing language across various note types. Table 2 presents the accuracy data for different note types, revealing notable variations in model performance. ED provider notes were the most accurate, achieving an overall accuracy of 69.4% and plan of Care notes had the lowest accuracy at 55.8%. When breaking down the accuracy by all types of variables in the final table, it was found that Plan of Care notes, processed by the 8B model with a temperature of 0.25 and with examples, achieved the highest accuracy at 78.9% (Figure 1). This was notably higher than the general accuracy of 55.8% for Plan of Care notes in Table 2.

**Table 2.** Performance by clinical note type.

Note type	Not stigmatizing				Stigmatizing				Overall accuracy (%)
	Predicted correct		Predicted incorrect		Predicted correct		Predicted incorrect		
	*N*	(%)	*N*	%	*N*	%	*N*	%	
ED provider notes	1616	84.2	304	15.8	366	39.1	570	60.9	69.4
Discharge summary	1301	86.7	199	13.3	306	37.0	522	63.0	69.0
Other	2720	86.2	436	13.8	629	35.4	1147	64.6	67.9
Consults	2279	85.2	397	14.8	819	36.9	1401	63.1	63.3
Progress notes	9817	83.1	1991	16.9	5078	36.9	8686	63.1	58.2
Plan of care	1093	86.7	167	13.3	655	35.0	1217	65.0	55.8

In addition to these quantitative findings, we reviewed a subset of notes where model and human annotations disagreed. Although a full qualitative error analysis was beyond the scope of this study, we include a brief thematic synthesis to acknowledge reviewer feedback. Misclassifications most commonly arose in long, clinically dense notes where neutral descriptions of complex illness or adverse events were mistaken by the models for judgmental language. For instance, medically appropriate descriptors related to disease severity (eg, controlled diabetes) or detailed explanations of fractures, chronic conditions, or postoperative complications were sometimes flagged as stigmatizing. Similarly, comprehensive emergency department or specialty notes that documented risk assessment was more likely to be misclassified due to their length and informational density.

## Discussion

The results of our analysis highlight several important trends regarding the impact of model size, temperature settings, and the inclusion of examples on the performance of pre-trained models in classifying stigmatizing language. Our findings reveal that a larger model size (8B) with examples performed best in terms of overall accuracy, which aligns with expectations. The increased capacity of these larger models allows them to process more complex language patterns and contexts,^42^ leading to higher accuracy in detecting stigmatizing language.

Temperature settings, however, exhibited more nuanced effects. The optimal temperature varied depending on the size of the model, suggesting that smaller models perform better with higher temperature settings, while larger models are more effective with lower temperature settings. Smaller models, with less parameterized weights, have a more limited ability to understand the full context of clinical notes. As a result, they benefit from higher temperatures, which introduce greater creativity and variability into the output. This increased randomness helps compensate for the model’s inability to fully grasp the complexities of the language, enabling it to make broader associations. On the other hand, larger models, with more parameterized weights and thus a richer knowledge base, perform better with lower temperature settings. The reduced randomness allows the model to rely more on its established knowledge and leads to more accurate, precise classifications. The results suggest that when knowledge is abundant, as in the case of larger models, excessive creativity (induced by higher temperature) can negatively impact accuracy by introducing noise and distorting the model’s ability to detect stigmatizing language. These findings are consistent with the work of Windisch et al, who also explored the impact of temperature settings on large language model performance, specifically for tasks like named-entity recognition and classification in clinical trial publications.^43^

This phenomenon may explain the struggles of the Deepseek R1 model. The intricacies of Deepseek’s “reasoning” remain largely unknown, particularly in untrained or poorly informed contexts, such as classifying clinical text as stigmatizing. The model’s middling performance highlights that its larger size (8B) combined with poorly grounded “reasoning” and examples behaves slightly better than the high-temperature (0.75) Llama 8B model without example-based guidance. The small improvement observed (∼2%) is likely due to the example-based guidance.

Our findings also provide insight into how the accuracy of pre-trained models in classifying stigmatizing language varied across different note types. Notably, ED provider notes achieved the highest overall accuracy at 69.4%, while Plan of Care notes exhibited the lowest accuracy at 55.8%. This discrepancy between note types suggests that certain note types may be more challenging for the models to process, potentially due to differences in structure, language complexity, or the nature of the content.

We also noted higher accuracy for Plan of Care notes in the context of the 8B model and lower temperature which highlights the importance of model configuration in overcoming challenges posed by specific note types. The Plan of Care notes may typically contain more formal, structured language, which could make it harder for the model to distinguish between stigmatizing and non-stigmatizing language without sufficient context. However, the inclusion of examples and the reduced temperature setting likely improved the model’s ability to detect these subtle distinctions, resulting in a higher accuracy. In contrast, the ED provider notes, which had the highest overall accuracy (69.4%), may contain more informal language and less structure, making them more straightforward for the model to process. This suggests that, in general, the models may be more effective at handling informal language or situations where the context is easier to infer, such as the fast-paced and dynamic nature of ED provider notes.

The qualitative findings help clarify why model performance varied across configurations. Many false positives reflected difficulty distinguishing between clinically appropriate descriptions of disease severity and language that conveys judgment or blame. These findings support the broader conclusion of this study that LLM performance in detecting stigmatizing language is sensitive to model settings and to the structure of clinical notes. Our results highlight the importance of careful evaluation and cautious interpretation when applying LLMs to real-world clinical documentation.

These findings have important implications for real-world applications of LLM models in clinical settings. They highlight the necessity of fine-tuning models to handle various note types effectively. In practice, a one-size-fits-all approach may not be sufficient; instead, models should be adapted to the specific characteristics of the clinical notes they are tasked with analyzing. This could involve adjusting parameters like temperature, model size, or even training on domain-specific examples to improve classification accuracy for various types of notes. Future work should explore whether fine-tuning or other model adaptation strategies could enhance performance by accounting for the distinct linguistic and structural characteristics of different clinical note types.

Our results highlight the importance of considering both the structure of the note and the optimal configuration of the model when aiming to achieve the highest accuracy in classifying stigmatizing language in clinical texts. Future work should focus on further refining these models, exploring ways to balance the performance across different note types, and ensuring that the models can be successfully deployed in diverse clinical contexts. Future studies could also expand the dataset to include a larger and more diverse set of clinical notes from multiple settings, as well as explore the impact of demographic factors (eg, age, gender, race) on model performance in detecting stigmatizing language. This could provide a more comprehensive understanding of how these models interact with different patient populations and the types of biases they may perpetuate.

### Implications for clinical practice

The implications of these findings extend broadly to clinical practice and healthcare delivery. Clinical documentation plays a central role in communication, decision-making, and patient care. Accurate identification and classification of stigmatizing language in electronic health records (EHRs) is essential to ensure that all patients are treated with dignity and respect, while also minimizing the potential impact of biased language on clinical decision-making and outcomes. Pre-trained models, when optimized for this task, could support healthcare professionals in detecting stigmatizing language in real time, enabling timely interventions and modifications to documentation practices. Models trained on a variety of clinical note types may also facilitate a deeper understanding of how language is used across settings (eg, emergency department vs care planning), thus enhancing consistency and equity in clinical communication.

Healthcare professionals bring critical expertise to the design and deployment of such tools. Their insight into the nuances of documentation practices and the clinical environment ensures that AI-driven solutions are both practical and contextually appropriate. Active engagement of clinicians in the development, implementation, and evaluation of these models will be essential for ensuring alignment with real-world workflows and patient-centered goals. Collaborative efforts between informatics experts, clinicians, and other stakeholders will be vital to the successful and ethical integration of these technologies into routine practice.

### Strengths and limitations

A key strength of this study is the comparison of model performance with that of human annotators. By benchmarking the models against human accuracy, we were able to see how closely the models align with expert judgment in identifying stigmatizing language. Our study also analyzed multiple note types, which adds depth to the analysis. This study is the first to assess the accuracy of large language models (LLMs) in classifying stigmatizing language across various types of clinical notes. This approach offers a more nuanced understanding of how model performance can vary based on the structure and language of different clinical documents.

This study has several limitations. We intentionally prioritized comparative evaluation of model configurations over optimization strategies. As such, we did not conduct iterative prompt engineering, perform example-order sensitivity analyses, or evaluate alternative prompt formats. While these approaches may improve performance in some settings, they were beyond the scope of the present work. Consequently, the findings should be interpreted as comparative rather than as validation of a production-ready system. Future work should explore prompt ablation, randomized ordering, and dynamic example selection to further evaluate the stability and generalizability of LLM performance in this context. We evaluated model performance using categorical outputs constrained to a single-token classification (“stigmatizing” vs “non-stigmatizing”). As a result, probabilistic confidence scores and token-level logits were not available, limiting our ability to conduct threshold-based evaluations such as receiver operating characteristic (ROC) or precision–recall (PR) analyses, calibration assessments, or computation of precision and F1 scores. Future research should prioritize the use of probabilistic outputs or logit-based scoring to enable more comprehensive performance evaluation, including precision, recall, F1, ROC, and PR analyses, as well as calibration testing. Our study did not also assess output stability across multiple runs of identical configurations, which would be important for evaluating reproducibility in high-stakes clinical applications. Future studies should incorporate repeated sampling and stability metrics to better understand model reliability. This study would be strengthened by a larger, more diverse sample drawn from multiple institutions across different regions of the United States. Expanding the list of LLMs tested and model sizes included in this study could have provided a broader perspective. Furthermore, a more extensive exploration of LLM parameter settings may yield deeper insights into their impact on the study’s outcomes.

## Conclusions

The findings from this study suggest that optimizing LLM models for detecting stigmatizing language in clinical documentation could be a valuable tool for addressing health disparities. However, careful attention must be given to model tuning, training data, and the integration of domain-specific knowledge to ensure that these tools are effective in real-world healthcare settings. For practice, this research highlights the need for continued collaboration between health care workers and AI developers to create tools that enhance communication, reduce bias, and improve the overall patient experience.

## Data Availability

The datasets generated and/or analyzed during the current study are not publicly available as it is electronic health records.
